# A gene locus for targeted ectopic gene integration in *Zymoseptoria tritici*^☆^

**DOI:** 10.1016/j.fgb.2015.03.018

**Published:** 2015-06

**Authors:** S. Kilaru, M. Schuster, M. Latz, S. Das Gupta, N. Steinberg, H. Fones, S.J. Gurr, N.J. Talbot, G. Steinberg

**Affiliations:** a Biosciences, University of Exeter, Exeter EX4 4QD, UK; b Geography, University of Exeter, Exeter EX4 4RJ, UK

**Keywords:** *sdi1*, succinate dehydrogenase 1, *sdhb*, succinate dehydrogenase subunit B, *Ip*, iron–sulfur protein, *sdi1*^R^, carboxin-resistant, GFP, green fluorescent protein, RFP, red fluorescent protein, eGFP, enhanced green fluorescent protein, AcGFP, *Aequorea coerulescens* green fluorescent protein, ZtGFP, *Z. tritici* codon-optimized green fluorescent protein, mCherry, monomeric cherry, TagRFP, monomeric red (orange) fluorescent protein, mRFP, monomeric red fluorescent protein, tdTomato, tandem dimeric red fluorescent protein, *tub2*, α-tubulin, RB and LB, right and left border, dpi, days post infection, ROI, region of interest, Succinate dehydrogenase, Dominant selectable marker, Wheat pathogenic fungi, *Septoria tritici* blotch, *Mycosphaerella graminicola*

## Abstract

Understanding the cellular organization and biology of fungal pathogens requires accurate methods for genomic integration of mutant alleles or fluorescent fusion-protein constructs. In *Zymoseptoria tritici*, this can be achieved by integrating of plasmid DNA randomly into the genome of this wheat pathogen. However, untargeted ectopic integration carries the risk of unwanted side effects, such as altered gene expression, due to targeting regulatory elements, or gene disruption following integration into protein-coding regions of the genome. Here, we establish the succinate dehydrogenase (*sdi1*) locus as a single “soft-landing” site for targeted ectopic integration of genetic constructs by using a carboxin-resistant *sdi1*^R^ allele, carrying the point-mutation H267L. We use various green and red fluorescent fusion constructs and show that 97% of all transformants integrate correctly into the *sdi1* locus as single copies. We also demonstrate that such integration does not affect the pathogenicity of *Z. tritici*, and thus the *sdi1* locus is a useful tool for virulence analysis in genetically modified *Z. tritici* strains. Furthermore, we have developed a vector which facilitates yeast recombination cloning and thus allows assembly of multiple overlapping DNA fragments in a single cloning step for high throughput vector and strain generation.

## Highlights

• We establish the *sdi1* of *Z. tritici* locus for targeted integration of constructs as single copies.
• Integration of constructs conveys carboxin resistance.
• We provide a vector for integration of eGFP-expressing construct into the *sdi1* locus.
• Integration into *sdi1* locus is not affecting virulence of *Z. tritici*.

## 1 Introduction

Homologous integration of genetic constructs into the genome of a fungus is a useful procedure to generate stable gene replacement mutants, but most DNA integration during fungal transformation proceeds by non-homologous, ectopic recombination. Such random integration of vector DNA into a fungal genome, however, has the potential to disrupt genes or introduce unwanted changes in gene regulation (Weld et al., 2006). Thorough examination of phenotypes of the resulting fungal transformations is recommended to reveal the presence of damaging insertional mutations. However, such analysis is labor-intensive and does not reveal more subtle changes that can be caused by random insertion of the vector DNA, such as small deletions or downstream effects on gene expression, or the capacity for tandem integrations of DNA leading to potential gene dosage problems with reporter gene constructs. These pitfalls can be circumvented by targeted integration of genetic constructs into a defined locus without disrupting other regions of fungal genomic DNA (Weld et al., 2006).

The systemic fungicide carboxin inhibits the mitochondrial succinate dehydrogenase (Ackrell et al., 1977; Tucker and Lillich, 1974). Succinate dehydrogenase consists of four subunits, and point mutations in these subunits confer resistance to carboxin in several fungi (Ito et al., 2004; Kilaru et al., 2009; Scalliet et al., 2012). Most commonly, a single point mutation in a histidine residue, located in the third cysteine-rich cluster of the SDHB subunit (gene name: *sdhb* or *sdi1* or *Ip*) has been used as selectable marker in fungi. This was initially established in the corn smut fungus *Ustilago maydis* (Broomfield and Hargreaves, 1992; Keon et al., 1991), and established in several other fungi, including *Zymoseptoria tritici* (former *Mycosphaerella graminicola*), *Pleurotus ostreatus*, *Lentinula edodes*, *Coprinopsis cinerea*, the ectomycorrhizal fungus *Hebeloma cylindrosporum*, several *Aspergillus* species, *Botrytis cinerea*, and *Ganoderma lucidum* (Honda et al., 2000; Irie et al., 2003; Kilaru et al., 2009; Ngari et al., 2009; Shima et al., 2009; Skinner et al., 1998; Yin et al., 2011; Yu et al., 2014).

Recently, Scalliet et al. (2012) generated various carboxin-resistant mutants of the wheat blotch fungus *Z. tritici* and reported that these strains are more virulent than control strains. Alterations in the virulence of carboxin-resistant mutants were also found in the maize pathogen *U. maydis* (Ruiz-Herrera et al., 1999), suggesting exploiting carboxin-resistance is not useful when investigating plant pathogenic fungi. However, subsequent studies in several carboxin-resistant mutants of *U. maydis* refuted these results, by showing no effect on pathogenicity (Topp et al., 2002). These results were confirmed by subsequent studies in *U. maydis*, showing that mutations in *sdi1* did not attenuate pathogenicity or, indeed, alter any cell biological processes (Bielska et al., 2014; Higuchi et al., 2014; Treitschke et al., 2010). Such findings encouraged us to revisit the use of the *Z. tritici sdi1* locus for targeted integration of vectors by using carboxin as the selection agent. We show that the *sdi1* locus is a useful site for targeted integration of constructs. We also demonstrate that integration of constructs into the *sdi1* locus by using carboxin has no detectable effect on virulence of *Z. tritici* on wheat. Finally, we provide a vector construct that combines yeast recombination-based cloning abilities with integration into the *sdi1* locus as single copy. This tool promises to be of significant utility for high throughput functional genomics studies in *Z. tritici*. To our knowledge, this is the first report utilizing the robustness of the yeast recombination cloning approach in combination with efficient targeted integration in the genome of *Z. tritici*.

## 2 Materials and methods

### 2.1 Bacterial and fungal strains and growth conditions

*Escherichia coli* strain DH5α was used for the maintenance of plasmids. *Agrobacterium tumefaciens* strain EHA105 (Hood et al., 1993) was used for maintenance of plasmids and subsequently for *A. tumefaciens*-mediated transformation of *Z. tritici. E. coli* and *A. tumefaciens* were grown in DYT media (tryptone, 16 g/l; yeast extract, 10 g/l; NaCl, 5 g/l; with 20 g/l agar added for preparing the plates) at 37 °C and 28 °C respectively. The fully sequenced *Z. tritici* wild-type isolate IPO323 (Goodwin et al., 2011; Kema and van Silfhout, 1997) was used as recipient strain for the genetic transformation experiments. The isolate was inoculated from stocks stored in NSY glycerol (Nutrient broth, 8 g/l; yeast extract, 1 g/l; sucrose, 5 g/l; glycerol, 700 ml/l) at −80 °C onto solid YPD agar (yeast extract, 10 g/l; peptone, 20 g/l; glucose, 20 g/l; agar, 20 g/l) and grown at 18 °C for 4–5 days.

### 2.2 Construction of targeted ectopic integration vector pCeGFP

**Fig. 1:**
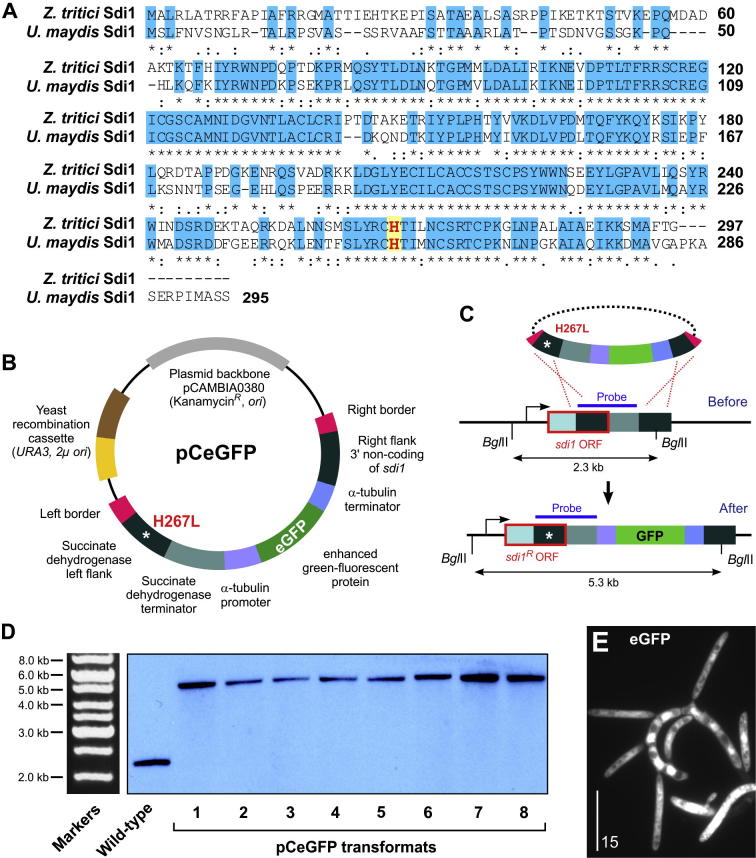
Establishing the *sdi* locus as a “soft-landing” site for targeted integration of vectors in to the genome of *Z.* tritici. (A) Comparison of the deduced amino acid sequences of succinate dehydrogenase subunit Sdi1 of *Z. tritici* and *U. maydis*. Identical amino acids are highlighted by blue background. Note that that the critical histidine at position 267 in *Z. tritici* is conserved (indicated in red). A mutation of this amino acid to leucine confers resistance to carboxin (Scalliet et al., 2012). (B) Schematic drawing showing the organization of vector pCeGFP. The fluorescent protein eGFP is expressed under the *Z. tritici* α-tubulin (*tub2*) promoter. After integration into the *sdi1* locus, the vector confers carboxin resistance due to a point mutation in the succinate dehydrogenase gene *sdi1*, which changes a histidine to leucine (H267L). Left and right border enable *Agrobacterium tumefaciens*-based transformation of *Z. tritici*. Note that fragments are not drawn to scale. For more accurate information on fragment sizes see main text. (C) Image illustrates the integration event of vector pCeGFP into the native *sdi1* locus of *Z. tritici*. This co-integrates a carboxin-resistant *sdi1*^H267L^ allele and cytoplasmic eGFP, expressed under the *tub2* promoter. (D) Southern blot showing integration of pCeGFP into numerous strains. Note that single integration into the desired locus was found in all carboxin-resistant transformants. The size markers in the corresponding agarose gel are shown to the left. (E) Image showing cytoplasmic eGFP expression in yeast-like cells of *Z. tritici* after integration of pCeGFP in to the *sdi1* locus. Bar represents 15 μm.

The vector pCeGFP was generated by *in vivo* recombination in the yeast *Saccharomyces cerevisiae* DS94 (MATα, *ura3-52*, *trp1-1*, *leu2-3*, *his3-111*, and *lys2-801* (Tang et al., 1996) following published procedures (Raymond et al., 1999; Kilaru and Steinberg, 2015). For all the recombination events, the fragments were amplified with 30 bp homologous sequences to the upstream and downstream of the fragments to be cloned (see Table 1 for primer details). The vector pCeGFP contains *egfp* under the control of *Z. tritici* α-tubulin promoter for integration in to the *sdi1* locus by using carboxin as a selection agent. A 9760 bp fragment of pCGEN-YR (digested with *Xba*I and *Zra*I), 706 bp of exon 3 of *sdi1* gene (amplified with SK-Sep-10 and SK-Sep-11; Table 1), a point-mutated (H267L) 308 bp fragment covering the last 111 bp of 3′ end *sdi1* gene and 197 bp downstream of the *sdi1* gene (amplified with SK-Sep-12 and SK-Sep-13; Table 1), 1149 bp *Z. tritici* α-tubulin promoter (amplified with SK-Sep-14 and SK-Sep-15; Table 1), 717 bp *egfp* (amplified with SK-Sep-16 and SK-Sep-78; Table 1), 1086 bp α-tubulin terminator (amplified with SK-Sep-162 and SK-Sep-19; Table 1) and 889 bp covering the right flank of *sdi1* gene (amplified with SK-Sep-25 and SK-Sep-26; Table 1) were recombined in yeast *S. cerevisiae* to obtain the vector pCeGFP (Fig. 1B). PCR reactions and other molecular techniques followed standard protocols (Sambrook and Russell, 2001). All restriction enzymes and reagents were obtained from New England Biolabs Inc (NEB, Herts, UK).

### 2.3 *Z. tritici* transformation

*A. tumefaciens* mediated transformation *of Z. tritici* was performed as described previously (Zwiers and De Waard, 2001), with the slight modifications. The vector pCeGFP was transformed into *A. tumefaciens* strain EHA105 by heat shock method (Holsters et al., 1978) and transformants were selected on DYT agar medium supplemented with 20 μg/ml rifampicin and 50 μg/ml kanamycin Sigma–Aldrich, Gillingham, UK). The obtained *A. tumefaciens* transformants were further confirmed by colony PCR and grown in 10 ml DYT medium supplemented with 20 μg/ml rifampicin (Melford, Ipswich, UK) and 50 μg/ml kanamycin for overnight at 28 °C with 200 rpm. The overnight cultures were diluted to an optical density of 0.15 at 660 nm in *Agrobacterium* induction medium AIM (10 mM KH_2_PO_4_, 10 mM K_2_HPO_4_, 2.5 mM NaCl, 2 mM MgSO_4_⋅7 H_2_O, 0.7 mM CaCl_2_, 9 mM FeSO_4_, 4 mM (NH4)_2_SO_4_, 10 mM glucose, 0.5% glycerol, 40 mM MES buffer, 1 l H_2_O, pH 5.6, with 20 g agar added for preparing the plates) supplemented with 200 μM acetosyringone (Sigma–Aldrich, Gillingham, UK) and grown at 28 °C with 200 rpm until an optical density reached to 0.3–0.35 (4–5 h). The *A. tumefaciens* cultures which contain the desired vectors were then mixed with an equal volume of *Z. tritici* yeast-like cells, which had been harvested from 5 day old YPD plates and diluted to a concentration of 1 × 10^8^ /ml in *Agrobacterium* induction medium. 200 μl of the *A. tumefaciens*–*Z. tritici* mixtures were plated onto nitrocellulose filters (AA packaging limited, Preston, UK) placed on AIM agar plates supplemented with 200 μM acetosyringone and grown at 18 °C for 3 days. Nitro cellulose filters were then transferred onto Czapek Dox agar plates (Oxoid, Basingstoke, UK) containing 100 μg/ml cefotaxime (Melford, Ipswich, UK), 100 μg/ml timentin (Melford, Ipswich, UK) and 40 μg/ml carboxin (Sigma–Aldrich, Gillingham, UK) and incubated at 18 °C until the colonies appear (for 8–12 days). The individual colonies were transferred on to YPD agar plates containing 100 μg/ml cefotaxime, 100 μg/ml timentin and 40 μg/ml carboxin and grown at 18 °C for 3–4 days.

### 2.4 Molecular analysis of transformants

To confirm the integration of vector pCeGFP into the *sdi1* locus of *Z. tritici* and also to determine the copy number, Southern blot hybridizations were performed by using the standard procedures (Sambrook and Russell, 2001). *Z. tritici* was grown in YG broth (yeast extract, 10 g/l; glucose, 30 g/l) for 3 days at 18 °C with 200 rpm and genomic DNA was isolated as described in Kilaru and Steinberg (2015). 3 μg of genomic DNA of IPO323 and transformants obtained with pCeGFP were digested with *Bgl*II and separated on a 1.0% agarose gel and capillary transferred to a Hybond N^+^ membrane (Life Science Technologies, Paisley, UK). 1014 bp *sdi1* probe (3′ end of the *sdi1^R^* gene and *sdi1*terminator) was generated with primers SK-Sep-10 and SK-Sep-13 (Table 1) by using DIG labeling PCR mix (Life Science Technologies, Paisley, UK). Hybridizations were performed at 62 °C for overnight and auto-radiographs were developed after an appropriate time period. This confirmed that the vector pCeGFP was successfully integrated in to the *sdi1* locus *of Z. tritici* and resulting in strain IPO323_CeGFP.

### 2.5 Microscopy

Fluorescence microscopy was performed as previously described (Schuster et al., 2011). Fungal cells were grown in YG medium (yeast extract, 10 g/l; glucose, 30 g/l) at 18 °C with 200 rpm for ∼24 h and placed onto a 2% agar cushion and directly observed using a motorized inverted microscope (IX81; Olympus, Hamburg, Germany), equipped with a PlanApo 100×/1.45 Oil TIRF (Olympus, Hamburg, Germany). eGFP was exited using a VS-LMS4 Laser Merge System with solid-state lasers (488 nm/50 mW or 75 mW and 561 nm/50 mW or 75 mW; Visitron Systems, Puchheim, Germany), and images were acquired using a CoolSNAP HQ2 camera (Photometrics/Roper Scientific, Tucson, USA). All parts of the system were under the control of the software package MetaMorph (Molecular Devices, Wokingham, UK).

### 2.6 Plant infection assays

Attached wheat leaf infections were performed as described previously (Rudd et al., 2008) with few modifications. Wheat cultivar Galaxie (Fenaco, Bern, Switzerland) was used for all the plant infections. Wheat seeds were sown in pots containing compost (John Innes Research centre, Norwich, UK) and grown at 22 °C with 500 μmol light (day) and 18 °C with 0 μmol light (night) with 60–65% humidity for 14 days (up to 10 plants/pot) *Z. tritici* strains (IPO323; IPO323_CAcGFP; IPO323_CeGFP; IPO323_CZtGFP, IPO323_CmRFP, IPO323_CTagRFP, IPO323_CmCherry and IPO323_CtdTomato) were grown on YPD agar plate at 18 °C for 5 days and the yeast-like cells were harvested and suspended in water and adjusted to a cell density of 1 × 10^6^/ml. The second leaves of 14-day old wheat seedlings were inoculated evenly with fungal spores at a density of 1 × 10^6^/ml in water containing 0.1% (v/v) Tween 20 (Sigma–Aldrich, Gillingham, UK) using paint brush. Plants were allowed to dry and then covered with transparent bags to retain high humidity and prevent cross contamination for the first 72 h. Plants were incubated at 22 °C (day) and 18 °C (night) with 60–65% humidity for additional 18 days.

### 2.7 Quantitative virulence analysis

Evaluation of symptoms was performed 16 days and 21 days after inoculation. At 16 dpi the infected leaves were scanned using an EPSON PERFECTION V750 PRO professional photo scanner (Epson, Hemel Hempstead, UK) and the percentage of 2nd leaf area covered with pathogen-related necrosis symptoms was analyzed in MetaMorph (Molecular Devices, Wokingham, UK). Using these digital images, the area of the entire leaf was measured. Secondly, the drawing tool was used to determine necrotic areas, defined as a color switch from green to yellow or brown. All measurements were transferred into Excel (Microsoft, Redmond, WA, USA) and the percentage of 2nd leaf area covered with necrosis symptoms was calculated. At 21 dpi infected leaves were imaged using a Nikon D500 SLR camera attached to a Nikon SMZ800 dissecting microscope (Nikon, Surrey, UK). The number of pycnidia developed after 21 dpi were analyzed using a high-throughput automated image analyzes macro for ImageJ, as described in Stewart and McDonald (2014).

## 3 Results and discussion

### 3.1 Identification of ZtSdi1

The *Z. tritici sdi1* (alternative name: *Ip*) gene along with 800 bp upstream and 300 bp downstream regions was first cloned and sequenced by Skinner et al. (1998). In order to establish *sdi1* as “soft-landing” site, further upstream and downstream sequences are required and we therefore screened the published genome sequence of Z. *tritici* (http://genome.jgi.doe.gov/Mycgr3/Mycgr3.home.html) with the predicted amino acid sequence of the succinate dehydrogenase subunit Sdi1 from *U. maydis* (UmSdi1; Broomfield and Hargreaves, 1992; XP_756991.1; obtained from http://www.ncbi.nlm.nih.gov/pubmed/). We identified the *Z. tritici* Sdi1 (ZtSdi1; protein ID 74146, accession number: XP_003850753.1). UmSdi1and ZtSdi1 share 57.5% sequence identity and 69.4% similarity. Like the enzyme in *U. maydis*, ZtSdi1 is predicted to form a 2Fe–2S iron–sulfur cluster binding domain (*P* = 2.8e−32) and a 4Fe–4S dicluster domain (*P* = 3.8e−10). Both proteins contain a histidine residue in the C-terminal half of their sequence (Fig. 1A, indicated in red) that, when mutated to leucine, confers resistance to the fungicide carboxin (Skinner et al., 1998).

### 3.2 A vector for targeted integration of constructs in to the *sdi1* locus

We used the identified *sdi1* sequence to construct a vector, pCeGFP, which allows targeted integration of DNA into the genomic locus of the succinate dehydrogenase subunit-encoding gene. The vector pCeGFP contains the gene encoding enhanced green fluorescent protein (*egfp*) under the control of constitutive *Z. tritici* α-tubulin promoter and terminator sequences (see Schuster et al., 2015b, for *tub2* promoter). The most important characteristic feature of this vector is that the presence of succinate dehydrogenase locus left flank and right flanks. The succinate dehydrogenase locus left flank is made up of 814 bp of 3′ end of *sdi1* gene (the full-length gene is 1005 bp) with H267L point mutation and 197 bp *sdi1* terminator (Fig. 1B, left flank); the right flank contains 889 bp sequence stretch downstream of *sdi1* (Fig. 1B, right flank). The point mutation, histidine to leucine (H267L), in the *sdi1* left flank confers resistance to carboxin after integration into the native *sdi1* locus (Fig. 1C). As the left flank contains only the partial *sdi1* gene along with H267L mutation, integration by homologous recombination mutates the endogenous *sdi1* gene (Fig. 1C, mutation indicated by asterisk) and thus only those transformants which undergo homologous recombination will survive on carboxin containing media. The 889 bp sequence stretch downstream of *sdi1* (right flank) also recombines endogenous 3′ non-coding sequence of *sdi1* and thus integrates the “payload” of the vector (Fig. 1C). In this instance the “payload” is the *egfp* gene under the control of *Z. tritici* α-tubulin promoter and terminator sequences.

In addition, this vector also carries a “yeast recombination cassette” consisting of URA3 and 2μ *ori* which enables cloning multiple overlapping DNA fragments in one step by using *in vivo* recombination in yeast without depending on the availability of restriction sites (Dagdas et al., 2012; Gibson et al., 2008; Kilaru et al., 2009; Ma et al., 1987; Schuster et al., 2011). This method keeps costs low, enables precise cloning of multiple fragments in a single step and, most importantly, avoids changes in the primary DNA sequence (Kilaru and Steinberg, 2015).

The vector pCeGFP was constructed by *in vivo* recombination in *S. cerevisiae* (for details see material and methods)*.* The *egfp* gene was cloned under the control of *Z. tritici* α-tubulin promoter and terminator sequences. These three fragments, the right flank of *sdi1* and the left flank of *sdi1*, containing a point mutation (H267L), were assembled in a single cloning step. The promoter and terminator sequences contain *Zra*I and *Mlu*I as unique restriction sites. These sites allow linearization of this vector for exchange of promoter and terminator sequences and/or cloning of additional DNA fragments. Using this approach, a broad range of additional vectors, containing fluorescent markers and tags, were generated (Kilaru et al., 2015a,b; Schuster et al., 2015a,b).

Molecular analysis of *Aspergillus awamori*, *M. oryzae* and *U. maydis* transformants via *A. tumefaciens* mediated transformation revealed single copy integrations with 87.5%, 72.6% and 96% frequencies, respectively (de Groot et al., 1998; Ji et al., 2010; Li et al., 2007). This is due to the efficient targeting of constructs, containing homologous sequences, by *A. tumefaciens* mediated transformation (Bundock et al., 1995). *A. tumefaciens*-mediated transformation for *Z. tritici* was successfully established previously (Zwiers and De Waard, 2001). Thus, we considered *A. tumefaciens*-mediated transformation ideal to establish the *sdi1* locus as “soft-landing” site with single copy insertions. The vector pCeGFP carries a kanamycin resistance gene, and origin of replications for *E. coli* and *A. tumefaciens*, which allows *A. tumefaciens*-based transformation into *Z. tritici*, based on the 25 bp imperfect directional repeat sequences of the T-DNA borders (right and left border, RB and LB; Fig. 1B). We therefore were able to use this method to target efficiently the DNA of interest into the *sdi1* locus of *Z. tritici.*

### 3.3 Targeted integration of vector pCeGFP in to the *sdi1* locus

We next set out to confirm the efficiency of targeted integration of vector pCeGFP into the *sdi1* locus of *Z. tritici.* To this end, we transformed vector pCeGFP into *Z. tritici* strain IPO323 (Kema and van Silfhout, 1997). The transformants were selected on Czapek Dox agar medium containing carboxin (for details see materials and methods). In order to confirm the integration of vector pCeGFP in to the *sdi1* locus of *Z. tritici*, the genomic DNA was purified from eight randomly selected transformants, and the wild-type isolate IPO323 included as control. The genomic DNA was digested with *Bgl*II and hybridised with *sdi1* probe (Fig. 1C; see materials and methods). In Southern blot of genomic DNA of all transformants, we found a single band at the expected size (5.3 kb; Fig. 1D) confirming integration of the vector pCeGFP into the *sdi1* locus in all cases. We extended this targeted integration analysis using various other constructs (Kilaru et al., 2015a,b; Schuster et al., 2015a,b). We found that integration into the *sdi1* locus as single copy occurred in 97% of all cases (28 constructs, in total 71 transformants). In the remaining 3% of the transformants, we found a second integration event had occurred in addition to the targeted *sdi1* locus integration (not shown). Thus, the *sdi1* locus is an efficient target for ectopic integration of genetic constructs. Finally, the vector pCeGFP allows expression of enhanced green fluorescent protein in the cytoplasm of *Z. tritici* (Fig. 1E).

### 3.4 Pathogenicity of carboxin-resistant *Z. tritici* transformants

**Fig. 2:**
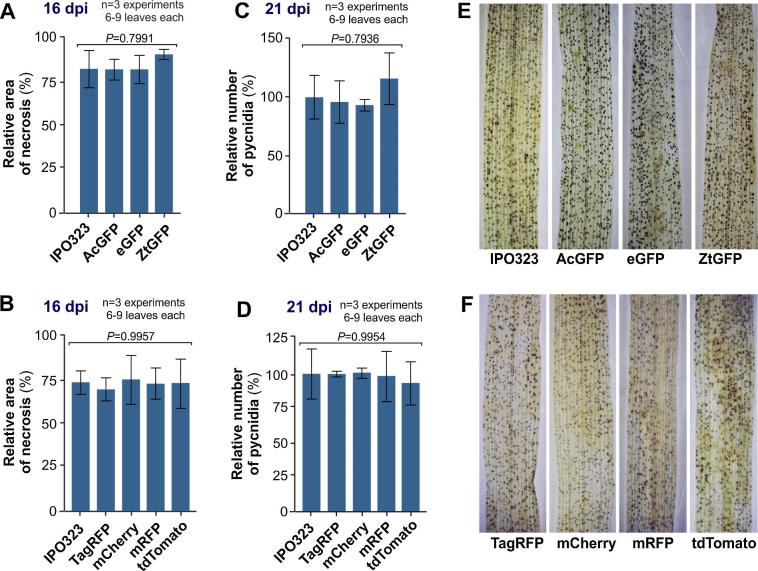
Pathogenicity of wild-type and carboxin-resistant *Z. tritici* strains, carrying various fluorescent proteins in their *sdi1* locus. (A) Bar chart showing the degree of necrosis on infected wheat leaves infected, at 16 dpi, with *Z. tritici* strains carrying green-fluorescent proteins in their *sdi1* locus. One-way ANOVA testing indicates no difference between wild-type IPO323 (Control) and strains expressing *A. coerulescens* GFP (AcGFP), enhanced GFP (eGFP) and *c*odon-optimized GFP for usage in *Z. tritici* (ZtGFP; error probability *P* = 0.7991; for more information on the strains and ZtGFP see Kilaru et al., 2015b). Mean ± SEM is shown, sample size *n* is indicated. (B) Bar chart showing the degree of necrosis on infected wheat leaves infected, at 16 dpi, with *Z. tritici* strains carrying red-fluorescent proteins in their *sdi1* locus. One-way ANOVA testing indicates no difference between wild-type IPO323 (Control) and strains expressing the red-fluorescent *Discosoma*-derived proteins monomeric RFP (mRFP), mCherry and tdTomato, as well as TagRFP from the sea anemone *E. quadricolor* (error probability *P* = 0.9957; for more information on the strains see Schuster et al., 2015a), Mean ± SEM is shown, sample size *n* is indicated. (C) Bar chart showing average pycnidia count per μm^2^ of infected leave area at 21 dpi. One-way ANOVA testing indicates no difference between wild-type IPO323 (Control) and strains expressing various GFPs (GFP from *A. coerulescens*: AcGFP, enhanced GFP from *A. victoria*: eGFP, enhanced GFP from *A. victoria*, codon-optimized for usage in *Z. tritici*: ZtGFP; error probability *P* = 0.7936). Mean ± SEM is shown, sample size *n* is indicated. (D) Bar chart showing average pycnidia count per μm^2^ of infected leave area at 21 dpi. One-way ANOVA testing indicates no difference between wild-type IPO323 (Control) and strains expressing various RFPs (monomeric red fluorescent protein, including TagRFP, generated from the wild-type RFP from sea anemone *E. quadricolor*), and various derivatives of the red fluorescent protein from *Discosoma* corals (mRFP, tdTomato and mCherry; error probability *P* = 0.9954). Mean ± SEM is shown, sample size *n* is indicated. (E) Wheat leaves at 21 days after infection with *Z. tritici* strains carrying various green fluorescent proteins in their *sdi1* locus. Brown dots represent fungal pycnidia. No obvious difference was found between wild-type IPO323 (Control) and strains expressing various GFPs (GFP from *A. coerulescens*: AcGFP, enhanced GFP from *A. victoria*: eGFP, enhanced GFP from *A. victoria*, codon-optimized for usage in *Z. tritici*: ZtGFP). More detail on the AcGFP and ZtGFP expressing strains can be found in Kilaru et al. (2015b). (F) Wheat leaves at 21 days after infection with *Z. tritici* strains carrying various red fluorescent proteins in their *sdi1* locus. Brown dots represent fungal pycnidia. No obvious difference was found between wild-type IPO323 (Control) and strains expressing various RFPs (monomeric red fluorescent protein, including TagRFP, generated from the wild-type RFP from sea anemone *E. quadricolor*), and various derivatives of the red fluorescent protein from *Discosoma* corals (mRFP, tdTomato and mCherry; for more detail on RFP-expressing strains see Schuster, et al., 2015a).

Previously, it has been reported that carboxin-resistant strains of the wheat blotch fungus are hyper-virulent (Scalliet et al., 2012). We re-tested this result using seven different carboxin-resistant IPO323-derived strains (IPO323_CAcGFP; IPO323_CeGFP; IPO323_CZtGFP, IPO323_CmRFP, IPO323_CTagRFP, IPO323_CmCherry and IPO323_CtdTomato; for detailed description of the strains see Schuster et al., 2015a), which express fluorescent fusion proteins from their *sdi1* locus. The fluorescent proteins included *Aequorea coerulescens* green-fluorescent protein (AcGFP; Gurskaya et al., 2003), enhanced GFP from *Aequorea victoria* (Yang et al., 1996) and a codon-optimised enhanced GFP from *A. victoria*, ZtGFP (Kilaru et al., 2015b). In addition, we tested strains expressing the red-fluorescent *Discosoma*-derived proteins mRFP (Campbell et al., 2002), mCherry and tdTomato (Shaner et al., 2004), as well as TagRFP from the bubble-tip sea anemone *Entacmaea quadricolor* (Merzlyak et al., 2007). All constructs were integrated into the *sdi1* locus as single copies as described in this paper. Firstly, we tested the degree of lesion coverage on wheat leaves of cultivar Galaxie. This assay is indicative of the susceptibility of a wheat cultivar to a given *Zymoseptoria* strain (Rosielle, 1972). We performed this analysis at 16 days post infection (16 dpi; for details the materials and methods), which is the time at which Scalliet et al. (2012) found increased virulence of *sdi1* mutant strains. By contrast, our analysis did not reveal any significant difference between wild-type control strain IPO323 and the three GFP-expressing carboxin-resistant strains (Fig. 2A; ANOVA testing, *P* = 0.7991). In a second round of pathogenicity assays we compared IPO323 against the four RFP expressing strains at 16 dpi. Again, we did not observe any difference in leaf necrosis (Fig. 2B; ANOVA testing, *P* = 0.9957). We next investigated the density of pycnidia at 21 dpi, which is an established method to assess virulence in *Z. tritici* (Eyal and Brown, 1976), using an automated recording procedure (Stewart and McDonald, 2014). Again, we did not detect any significant differences in symptom development between IPO323 and the seven carboxin-resistant strains (Fig. 2C–F; ANOVA testing, *P* = 0.7936 and 0.9954). We conclude that neither the modifications of the *sdi1* locus that results in carboxin-resistance, nor the cytoplasmic expression of the tested fluorescent proteins affects virulence in *Z. tritici*. But how do we therefore explain the discrepancy with the published information by Scalliet et al. (2012)? In our hands, all *Z. tritici* strains caused ∼75–85% necrotic leaf area (Fig. 2A and B). This degree of necrotic leaf area corresponds well to the data published for carboxin-resistant mutants by Scalliet and co-workers. However, in their study IPO323 showed only ∼25% necrosis. Consequently, all carboxin-resistant strains were therefore considered hyper-virulent (Scalliet et al., 2012). By contrast, in our study, as well as in other studies (Roohparvar et al., 2007), IPO323 was much more aggressive, reaching to ∼80% necrotic leaf area (Fig. 2A). Thus, one explanation could be that the control, used by Scalliet et al. (2012), did not develop full virulence, which could be due to spontaneous mutations of the strain utilized, or long term storage attenuating its virulence. We conclude there is no effect on virulence from the use of this selectable marker system.

## 4 Conclusion

We developed an ideal gene targeting system for *Z. tritici*, which allows precise integration of DNA of interest in to the *sdi1* locus without deleting or distributing fungal genes. The *sdi1* mutant allele itself will function as dominant selectable marker and thus this protocol avoids the use of bacterial dominant selectable markers. The robustness of the yeast recombination cloning in combination with high efficiency gene targeting system allows high-throughput functional genomics studies in *Z. tritici.* Furthermore, we have shown that generation of carboxin-resistant transforms of *Z. tritici*, it does not influence disease symptoms. Thus, we show that using the *sdi1* locus for targeted integration is a useful method to generate mutant strains in *Z. tritici*.

## Figures and Tables

**Table 1:** Primers used in this study.

Primer name	Direction	Sequence (5′–3′)^a^
SK-Sep-10	Sense	*TGGCAGGATATATTGTGGTGTAAACAAATT*GACCTTCCACATCTACCGATGG
SK-Sep-11	Antisense	*ATTCAGAATGGTGAGGCATCGGTACAAGCT*CATGCTGTTGTTGAGTGCGTCC
SK-Sep-12	Sense	*AGCTTGTACCGATGCCTCACCATTCTGAAT*TGCTCAAGGACCTGCCCCAAG
SK-Sep-13	Antisense	CTTCCGTCGATTTCGAGACAGC
SK-Sep-14	Sense	*CATTTGCGGCTGTCTCGAAATCGACGGAAG*GCAGTCGACGCCAGATGATGG
SK-Sep-15	Antisense	*GGTGAACAGCTCCTCGCCCTTGCTCACCAT*GGCGATGGTGGTATGCGGATG
SK-Sep-16	Sense	ATGGTGAGCAAGGGCGAGGAG
SK-Sep-19	Antisense	GAGGAGTCGACAGCCAAGCTC
SK-Sep-25	Sense	*CTCTCATAAGAGCTTGGCTGTCGACTCCTC*ACATTTTACAACATACTCAAGTCTG
SK-Sep-26	Antisense	*TAAACGCTCTTTTCTCTTAGGTTTACCCGC*GTTGAAGTTCTGCGTCGGATCC
SK-Sep-78	Antisense	*CCACAAGATCCTGTCCTCGTCCGTCGTCGC*TTACTTGTACAGCTCGTCCATGC
SK-Sep-162	Sense	GCGACGACGGACGAGGACAG

a *Italics* indicate part of the primer that is complementary with another DNA fragment, to be ligated by homologous recombination in *S. cerevisiae*.
